# ARCHIVES OF GERONTOLOGY AND GERIATRICS: MEN’S HEALTH AND AGING

**DOI:** 10.1093/geroni/igz038.779

**Published:** 2019-11-08

**Authors:** Roland J Thorpe

**Affiliations:** Johns Hopkins Bloomberg School of Public Health, Baltimore, Maryland, United States

## Abstract

Historically men typically have had more opportunities, privileges, and power; yet men die sooner and have earlier onset of health conditions compared to women. This is largely because there is a paucity of research focusing on the complex interaction that exists between social, behavioral, biological, and psychosocial factors among men. This symposium contains a collection of papers in the latest Volume of the Archives of Gerontology and Geriatrics that discuss some key factors that can provide insights to advance our understanding of men’s health and aging. Kelley and colleagues bring together several important concepts from sociology and gerontology to provide an explanatory framework for older men’s differential health profiles within and between cohorts, and over time. Wilmoth and colleagues discuss the complexities of understanding the health and well-being of male veterans in late life by providing critical insight on next steps that are needed on specific war-era cohorts to identify the mechanisms that shape veteran status differences in late-life health and mortality. Taylor and Taylor focus on social isolation and loneliness among a diverse sample of older men including understanding how social isolation and loneliness impact health outcomes. Bruce and Thorpe focus on how faith has implications for socio-biologic interactions associated with elevated risk for disease and premature death among this marginalized population. These presentations collectively will bolster our knowledge on men’s health and aging.

